# Buried or unburied K-wires for lateral condyle elbow fractures

**DOI:** 10.1308/003588412X13171221592375

**Published:** 2012-10

**Authors:** L McGonagle, S Elamin, DM Wright

**Affiliations:** Alder Hey Children’s NHS Foundation Trust,UK

**Keywords:** Lateral, Condyle, Fracture, K-wire, Burial

## Abstract

**INTRODUCTION:**

Lateral humeral condyle fractures typically require a longer period of internal fixation than other distal humeral fractures due to the increased risk of non-union. K-wires can be buried and left in situ until union or they can be left unburied and require removal after four weeks, with plaster immobilisation until union. There is no consensus as to whether wire burial is preferable or not. The aim of this study was to determine whether K-wire burial is associated with more complications than non-buried wires in treating lateral condyle fractures of the elbow.

**METHODS:**

All patients with lateral humeral condyle fractures treated with K-wire fixation at our institution from May 2008 to August 2011 were included in the study. Fracture configuration, mode of reduction, wire burial and complications were assessed.

**RESULTS:**

Sixty-seven patients (19 girls and 48 boys, mean age: 6.5 years, range: 1–17 years) were included in the study. All had closed injuries and were treated with open reduction and K-wire fixation. K-wires were buried in 55 patients. Thirteen cases of buried wires eroded through skin and were removed on average 45 days (range: 30–58 days) post-operatively. Of the wire erosion cases, three developed microbiologically proven infections, one of which was a deep infection. There were a further three superficial wound infections in the absence of wire erosion through the skin. There were complications in 2 of the 12 cases in the unburied wires group: 1 microbiologically proven superficial wire site infection and 1 wire backed out after 11 days, requiring refixation.

**CONCLUSIONS:**

Wire erosion through the skin is the most common complication of K-wire burial. This may be due to the decrease in swelling after fracture fixation, making the wires more prominent under the skin. Skin integrity should be monitored closely if wires are buried.

Lateral condyle fractures of the elbow are common injuries accounting for 17% of all distal humeral injuries in children.^1^ Displaced fractures often require open reduction and internal fixation to ensure that the intra-articular component has anatomical reduction. Internal fixation is attained with the use of K-wires although compression screws can be used in older children.^2–4^

There is no clear consensus as to whether K-wires should be buried or left exposed outside the skin, as is the case with supracondylar fractures of the distal humerus. The main theoretical argument for burying the wires is that exposed wires may be more prone to pin site infection and the presence of a superficial infection could track rapidly into the joint with devastating consequences. Unburied wires are often removed after about four weeks to minimise the risk of infection. Nevertheless, after wire removal, fracture healing may not have progressed sufficiently to allow unprotected motion. Further plaster immobilisation for another few weeks is therefore often required.^5^ The main benefit of wire burial is that the wires can be left in situ until clinical and radiographic evidence of fracture union is evident. However, a second general anaesthetic is necessary for wire removal.

In our institution we have noted problems with skin erosion with the use of buried K-wires. Given the controversy as to whether or not to bury K-wires, we aimed to compare the results and complications of buried and non-buried K-wires in the treatment of these fractures.

## Methods

**Figure 1:**
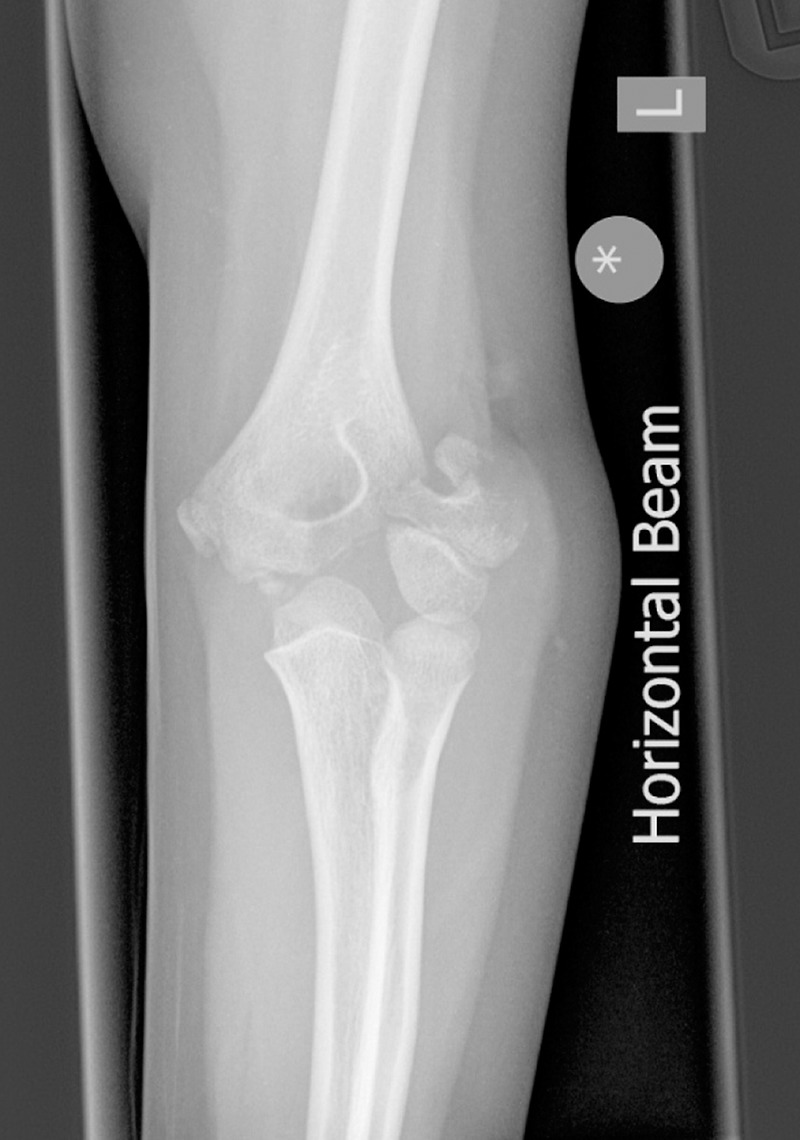
Elbow lateral condyle fracture (Milch type II)

All lateral condyle fractures of the distal humerus that were treated surgically from May 2008 to August 2011 were identified from the trust trauma database. Injury mechanism, fracture classification, time to surgery, open/closed reduction, operating surgeon grade, wire burial, wire orientation and post-operative complications were documented. Data were retrieved from case notes, the picture archiving and communication system radiology archive and the electronic operative notes archive. Patients with an elbow lateral condyle fracture (Fig 1) that was treated with K-wires were included in the study. Patients who were treated non-operatively or with screws were excluded.

There were 81 patients with lateral condyle fractures of the elbow. Five patients were treated non-operatively while seventy-six underwent surgery. Of these, 7 had internal fixation with screw(s) and were excluded, leaving 69 patients who had internal fixation with K-wires. There were 50 boys and 19 girls (mean age: 6.5 years, range: 1–17 years). All patients were followed up for an average of 18.7 weeks.

### Operative techniques

Sixty-seven cases were treated by open reduction and K-wire fixation. The wires were buried subcutaneously in 55 cases and unburied in 12 cases. Of the two closed reductions, intra-operative arthrography was used in one case to confirm reduction. Both cases were internally fixed with percutaneous divergent K-wires that were removed at seven weeks.

**Figure 2:**
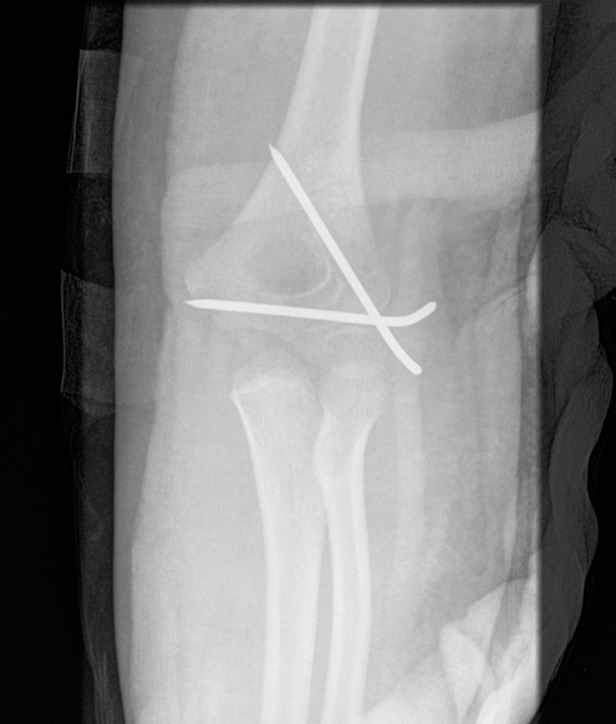
Divergent K-wire fixation of fracture, wires buried

Open reduction was carried out under general anaesthesia with fluoroscopic guidance and the use of a high tourniquet. The plane between the brachioradialis and the triceps was identified. The common extensor origin was partially mobilised to adequately visualise the joint surfaces. The articular surfaces were reduced anatomically and fixed with K-wires. Wire divergence was attained when possible (Fig 2). An above elbow backslab was applied with the elbow flexed to approximately 90º and the forearm in neutral rotation.

Patients were reviewed in clinic at one week with an up-to-date x-ray to confirm satisfactory position. The backslab was changed to a full above elbow plaster at this stage. There was no difference in plastering technique between the two groups. Further review at six weeks was planned to confirm fracture union and plan wire removal.

## Results

### Unburied wires

Of the twelve cases with unburied wires, there were three Milch type I fractures and nine Milch type II fractures. A consultant was the lead surgeon in four cases (one Milch type I, three Milch type II fractures), a trainee performed the other eight cases. In all but one of the cases, the wires were passed through a separate stab incision from the one used to openly reduce the fracture. All wires were passed in a divergent fashion and removed after an average of 6.5 weeks (range: 5.0–8.7 weeks).

#### Unburied wires – complications

One infection was noted after six weeks when the wires were removed. *Staphylococcus aureus* was cultured from the wound swab and treated with surgical wound washout and antibiotics. Furthermore, one wire backed out after 11 days, requiring refixation.

### Buried wires

Of the 55 cases with buried wires, there were four Milch type I fractures and 51 Milch type II fractures. The procedure was performed by a trainee in 49 cases and a consultant in 6 (all Milch type II fractures). The wires were inserted in a divergent fashion in 51 cases and parallel in 4 cases. Wires were removed at an average of 8 weeks (range: 4–20 weeks). In 3 cases wires were removed >12 weeks after surgery. All three were clinically and radiologically united at eight weeks. However, delay in wire removal was due to patient request in one case and two patients were listed as elective cases.

#### Buried wires –complications

> *Skin erosion*: Erosion through the skin was the most common problem and occurred in 13 cases. The mean time of removal was 6.4 weeks post-operatively. In 9 of the 13 cases, the wires were removed on the same day that erosion was noted. One patient’s wires were removed a week later for logistical reasons as he did not live locally. Three other cases had wire removal within two weeks as the fracture had not united satisfactorily at the time that skin erosion was noted. One of these patients had a wound infection that was treated with intravenous and oral antibiotics prior to wire removal.
> *Infection*: Of the buried wires that eroded through the skin, three cases had microbiologically confirmed infection. All were treated with antibiotics, which was sufficient in two cases. The third patient required two surgical debridements. Of the buried wires that did not erode through the skin, two developed a microbiologically confirmed wound infection. Both cases required antibiotics, one of which also needed surgical debridement.
> *Inadequate reduction*: This was noted on follow-up radiographs two weeks post-operatively. This case underwent revision fixation with no further problems.

No fracture in either group went on to non-union.

## Discussion

Pin site infections are a possible complication with any internal fixation. Supracondylar elbow fractures are often treated with non-buried wires. In these cases, the rate of deep infection is approximately 0.2% while that for superficial infection is 0.8%.^6^ The infection rate of lateral condyle fractures has been noted to be higher (8.1%) but in the study by Koh *et al* it is not clear what proportion of K-wires were buried.^7^ Some studies have directly compared buried and non-buried wires in lateral condyle fractures but no significant difference in outcome or complications was shown between the two groups.^8^

In our study the most common problem encountered was skin erosion (24%) in the buried wires group. This necessitated early wire removal compared with the buried wires, which did not erode the skin (6.5 weeks vs 8 weeks). This skin breakdown renders wire burial ineffective and also causes damage to the skin. We believe that this complication occurs because the soft tissue swelling evident immediately after the injury and at the time of surgery subsides several days after fixation. This results in the K-wires being more prominent, and thereby increasing the risk of skin breakdown.

One of the main (anecdotal) arguments for wire burial is to minimise the risk of superficial pin site infection, which, theoretically, may progress to septic arthritis as the wires are intra-articular. This theory is not supported by our data, which indicate that the infection rate was approximately 10% in each group. There were no cases of septic arthritis in either group.

There is a lack of data concerning whether it is preferable to bury K-wires in lateral condyle fractures. Chan and Siow compared the 2 treatment options in a retrospective cohort analysis of 42 buried wires versus 33 exposed wires.^8^ There was a low complication rate with one superficial infection and two cases of overgranulation, all in the exposed wires group. There were no cases of skin erosion in the buried wires group.

Thomas *et al* compared 97 exposed wires and 7 buried wires that were left in situ for 3 weeks.^9^ The wire related problems included: one case of wire erosion and septic arthritis in one of the buried wires cases, and one superficial infection in an exposed wires case. Launay *et al* had 57 displaced lateral condyle fractures, of which 32 had percutaneous K-wires.^10^ There were eight superficial infections and one deep infection in the percutaneous group. The subcutaneous group had two superficial infections, which, the authors propose, were due to excessive wire length irritating the skin. This led the authors to advise the use of subcutaneous wires. However, studies involving the hand and wrist indicate that unburied wires are associated with a higher rate of pin site infection when compared with buried wires.^11–13^

The benefit of longer internal fixation afforded with buried wires should be offset against the increased risk of skin breakdown. Skin integrity should be monitored closely if wires are buried.

## Conclusions

The idea behind burying K-wires is to reduce infection rates because it is felt that the wires have to stay in for a minimum of six weeks to prevent non-union. In this study there were no cases of non-union in either the buried or unburied wires group and infection rates were comparable. In addition, in 25% of cases the buried wires became effectively unburied. We therefore feel that there is no justification in burying wires, which requires an additional general anaesthetic, further burdening already stretched services. We now advocate leaving the wires outside the skin for 4–6 weeks and a minimum time in cast of 6 weeks until radiological evidence of union is seen.
